# Visual Processing of Biological Motion in Children and Adolescents with Attention-Deficit/Hyperactivity Disorder: An Event Related Potential-Study

**DOI:** 10.1371/journal.pone.0088585

**Published:** 2014-02-10

**Authors:** Anne Kröger, Katharina Hof, Christoph Krick, Michael Siniatchkin, Tomasz Jarczok, Christine M. Freitag, Stephan Bender

**Affiliations:** 1 Department of Child and Adolescent Psychiatry, Psychosomatics, and Psychotherapy, Goethe University Hospital Frankfurt, Frankfurt am Main, Germany; 2 Department of Neuroradiology, Saarland University Hospital, Homburg an der Saar, Germany; University of Tuebingen Medical School, Germany

## Abstract

Attention-deficit/hyperactivity disorder (ADHD) is often accompanied by problems in social behaviour, which are sometimes similar to some symptoms of autism-spectrum disorders (ASD). However, neuronal mechanisms of ASD-like deficits in ADHD have rarely been studied. The processing of biological motion–recently discussed as a marker of social cognition–was found to be disrupted in ASD in several studies. Thus in the present study we tested if biological motion processing is disrupted in ADHD. We used 64-channel EEG and spatio-temporal source analysis to assess event-related potentials associated with human motion processing in 21 children and adolescents with ADHD and 21 matched typically developing controls. On the behavioural level, all subjects were able to differentiate between human and scrambled motion. But in response to both scrambled and biological motion, the N200 amplitude was decreased in subjects with ADHD. After a spatio-temporal dipole analysis, a human motion specific activation was observable in occipital-temporal regions with a reduced and more diffuse activation in ADHD subjects. These results point towards neuronal determined alterations in the processing of biological motion in ADHD.

## Introduction

Attention-deficit/hyperactivity disorder (ADHD) is one of the most common disorders in child and adolescent psychiatry and shows a prevalence of about 5% in the general population [1], [2]. In addition to symptoms of inattention, hyperactivity and impulsivity, core symptoms of autism-spectrum disorders (ASD) including social communication and interaction deficits are frequently described in children and adolescents with a primary diagnosis of ADHD even though these symptoms are still below the cut-off for an ASD diagnosis [3]–[7]. Others only found elevated communication and social interaction deficits but no elevated repetitive behaviors [8]. However, these ASD-like symptoms seem to be a marker of a more strongly affected ADHD group with higher rates of comorbid disruptive behavior (including conduct and oppositional defiant disorder), other developmental disorders [5] and a higher co-occurrence of motor problems [9]. Furthermore these symptoms are clinically highly relevant for children with ADHD because especially deficits in the social domain are associated with a poor prognosis and higher risk for the development of other psychiatric problems, including mood disorders, anxiety, disruptive behavior disorders and substance abuse [10]. With regard to etiology and also with regard to specific therapeutic approaches, it is important to study if the clinically observed social interaction difficulties in ADHD might be associated with neural processing abnormalities.

Up to date, only a few experimental studies about social cognition deficits in ADHD can be found (for a review see [11]). Those few found e.g. disruptions in prosody perception and facial affect recognition. Reports about deficits in more complex tasks including theory of mind or empathy are more heterogeneous. Neuroimaging or neurophysiological studies about social cognition deficits in ADHD are even rarer. There is one neurophysiological study [12] which found several neural processes of emotional face processing to be disrupted in children with ADHD, including a reduced neuronal activation during early perceptual analysis (amplitude of the P120), elevated activity during processes associated with perceptual analysis of emotional expression (N170 amplitude), followed by a reduced activation in the P300, a marker for context processing. However these processes were partly normalized after treatment with methylphenidate.

Processing of biological motion, including human motion patterns (often presented in point-light displays), has recently been discussed as a marker of social cognition [13]. Indeed recognition of biological motion, such as eye movements and body gestures, is crucial for the development of adequate social interaction and adaptive responses, and usually emerges early in development [14], [15]. In addition, one recent study [16] found correlations between measures of social perception (empathy, emotion recognition and theory of mind) and the individual ability in using form cues in biological motion processing in typically developing controls. The ability to detect emotions in point-light displays of a walking human correlates furthermore with the ability to discriminate biological from scrambled motion patterns [17]. A similar pattern was found in a sample of subjects with ASD [18]. In functional magnetic resonance imaging (fMRI) studies, neuronal structures in the right posterior temporal cortex were identified to be involved in the processing biological motion, especially the posterior superior temporal sulcus (pSTS) [13], [19]. These structures are furthermore also important for the processing of socially relevant visual information e.g. emotions expressed by whole body movements and intentions of others revealed by actions and movement [20], [21]. Especially in ASD, abnormal brain activation patterns to biological motion stimuli have been found, predominantly by fMRI studies (for a review see [22]).

Biological motion processing can also be studied with electroencephalography (EEG). In contrast to fMRI, EEG can differentiate time related sub-processes of visual processing. Regarding biological motion, first, a positive component 100 ms after stimulus onset can be observed at occipital electrodes, especially in the right hemisphere [23], [24]. This P100 component is thought to evolve from the visual areas V1 and V2 [25] and to reflect stimulus feature extraction, including contrast, luminance, motion detection [26], [27], and pattern processing [28], [29]. After this first component, a negative deflection peaking around 200 ms is frequently described [23], [30]–[32]. As the source of the N200 is close to MT+/V5, it seems to reflect, more specifically, motion processing [32] including detection of motion direction [27]. Using high-density electrical mapping and source-analysis on difference waves (biological – scrambled motion), Krakowski et al. described a dipolar activity after approximately 300 ms. Its source was located in posterior middle temporal regions, near the pSTS [24], a neuronal region especially involved in the processing of human motion [19] and also social information [33]. Finally, a positive deflection at centro-parietal electrodes starting approximately after 400 ms was described, with greater amplitudes during human motion processing when the human motion aspect of the stimuli was actively attended [24]. This component is thought to be a marker of top-down cognitive processes involved in active decoding of stimulus content and could be a sub-component of the so-called late positive complex (LPC). A particularly late and broadly distributed LPC around 500–600 ms was also observed in other studies, when object parts are poorly specified by the available contours and thus difficult to identify [34].

In the present study, we tested if biological motion processing is disturbed in children and adolescents with ADHD. To our knowledge there are no studies about biological motion processing or perception in ADHD. Therefore we assessed event-related potentials (ERPs) elicited by biological and scrambled motion to test if biological-motion processing is abnormal in these participants. More elevated ASD-symptoms and thus more social deficits were previously found in children with ADHD and comorbid disruptive behaviour disorders [5]. Therefore we compared ADHD patients with and without conduct disorder (CD) or oppositional defiant disorder (ODD) to assess the effects of a comorbid diagnosis of CD/ODD. We additionally tested for correlations between ADHD- and ASD-symptoms with brain activity related to disturbed biological motion processing to further test if such deficits are more related to ADHD or subclinical ASD symptoms.

## Methods

### Participants and recruitment

We included 48 children and adolescents in our study. Two participants had to be excluded because they showed too few responses during the experiment (one typically developing control and one ADHD participant), and four because of muscle artefacts in the EEG data (two in each group). The remaining 21 typically developing controls and 21 children and adolescents with ADHD were 7 to 15 years old (see Table 1). Only male and right-handed children with normal or corrected-to-normal vision were included. Handedness was validated using the Edinburgh Handedness Inventory [35].

**Table 1:** Descriptive data for typically developing controls (TC) and ADHD subjects.

Mean (±SD)	TC (N = 21)	ADHD (N = 21)	statistics
Range (min–max)			
Age	11.64 (±2.42)	11.94 (±1.75)	t(36.4)<1; *p = 0.6*
	7.67–15.27	8.76–14.81	
Handedness	0.84 (±0.14)	0.79 (±0.18)	z<1; *p = 0.4*
	0.54–1	0.50–1	
Raven Percentile	59.10 (±38.16)	71.19 (±29.19)	z<1; *p = 0.5*
	3–100	3–100	
SCQ score	(N = 19*) 4.11 (±3.07)	(N = 20*) 7.45 (±4.78)	t(32.6) = −2.1; *p = 0.014*
	0–12	1–16	
Correct responses walker condition	84.81 (±6.78)	85.29 (±6.15)	
	66–90	64–89	
Correct responses scramble condition	83.57 (±4.28)	82.24 (±9.68)	Group: F(1,39)<1; *p = 0.77*
	61–90	59–89	
Reaction time walker condition in sec	1.35 (±0.29)	1.43 (±0.21)	
	0.76–1.89	0.82–1.66	
Reaction time scramble condition in sec	1.42 (±0.28)	1.51 (±0.24)	Group: F(1,39)<1; *p = 0.35*
	0.79–1.75	0.83–1.75	

*different sample sizes because of missing questionnaires.

ADHD out-patients were diagnosed according to ICD-10 (F90.0; [36]) at the Department of Child and Adolescent Psychiatry of the Goethe University of Frankfurt (Germany) by experienced clinicians. The ICD-10 diagnosis F90.0 correspondents to the ADHD combined subtype as described in DSM-IV TR (314.01; [2]), but hyperactive-impulsive symptoms were rated according to ICD-10. Before participating in the study, ADHD and comorbid diagnoses were verified with a semi-structured interview according to ICD-10 criteria (Kinder-DIPS; [37]). Thirteen subjects diagnosed with ADHD were treated with the psycho-stimulant methylphenidate. Methylphenidate inhibits the reuptake of dopamine and norepinephrine into the presynaptic neuron following their release. Thus, it increases levels or prolongs availability of these neurotransmitters in the synapses to exert its effects on postsynaptic neurons (e.g. [38]–[40]). Nine subjects received extended release methylphenidate, which was stopped at least 48 hours prior to participation. Four subjects were medicated with immediate release methylphenidate, which had not been taken for at least 24 hours before participation. Last application of medication varied between 24–252 hours (immediate release methylphenidate) and 48–228 hours (extended release methylphenidate).

Typically developing controls were recruited from local schools and screened with the German version of the Child Behaviour Checklist [41] for any clinically relevant symptoms. Participants with T-scores>60 on the second order scales or >70 on any first order subscales, respectively, were excluded.

Exclusion criteria for both groups were as follows: intellectual disability according to a standardized IQ assessment (percentile rank<2 corresponding to IQ<70 respectively; for details see below), any neurological disorders (including epilepsy), preterm birth with low birth weight (<2000 g), and dyslexia. In children with ADHD, comorbid psychiatric disorders (e.g. ASD, anxiety disorders) were excluded with the exception of oppositional defiant disorder (ODD) and conduct disorder (CD). Nine boys with ADHD and comorbid ODD or CD (ICD-10: F90.1) were included.

Subjects received a small fee for their participation. All participants and parents signed informed consent. The study was approved by the Ethics Committee of the Goethe University Frankfurt (Germany) in accordance with the Declaration of Helsinki.

### Psychological assessment

IQ was assessed using the standard (over 11.5 years) or coloured (under 11.5 years) progressive matrices [42], [43]. The matrices test is a non-verbal, multiple-choice IQ assessment tool which measures deductive reasoning. Raw scores are transformed into percentiles corresponding to the respective age group.

Autistic symptoms were assessed by the German version of the Social Communication Questionnaire (SCQ; [44]) in all participants. This questionnaire was developed as a companion tool to the ADI-R [45] and shows good psychometric properties. The SCQ consists of 40 yes/no questions answered by the participants' main caregivers. To differentiate ASD from typically developing controls a cut-off = 17 was suggested in a German sample [46].

In ADHD subjects, ADHD symptoms were assessed with a parent rating scale taken from a German diagnostic system for mental disorders in children and adolescents (DISYPS-II; [47]). In this questionnaire, each DSM-IV TR derived symptom is rated on a scale between 0–3, with a score of 3 indicating the most severe problems. Parents from the ADHD group were asked to rate the behaviour of their child without medication. A total score and sub-scores for attention problems (9 items) and hyperactive/impulsive behavior (11 items) were calculated.

The SCQ and the ADHD symptom checklist were used to assess the level of these traits/symptoms in both groups and to be able to test if brain activity related to disturbed biological motion processing is related with ADHD or subclinical ASD symptoms.

Both questionnaires were not returned by all participants. The SCQ was missing in two typically developing controls and in one ADHD subject. The ADHD symptoms rating scale was missing in one ADHD subject. Those subjects were excluded from calculations regarding the respective questionnaire.

### Experimental design, procedure and stimulus presentation

A 30×37.5 cm flat-screen was placed 80 cm in front of the head of the participant. In order to keep this distance constant and to minimise head movement, the participants had to place their chins on a ‘chin-rest’ fixed to the table. The room was dark during the entire experiment.

**Figure 1:**
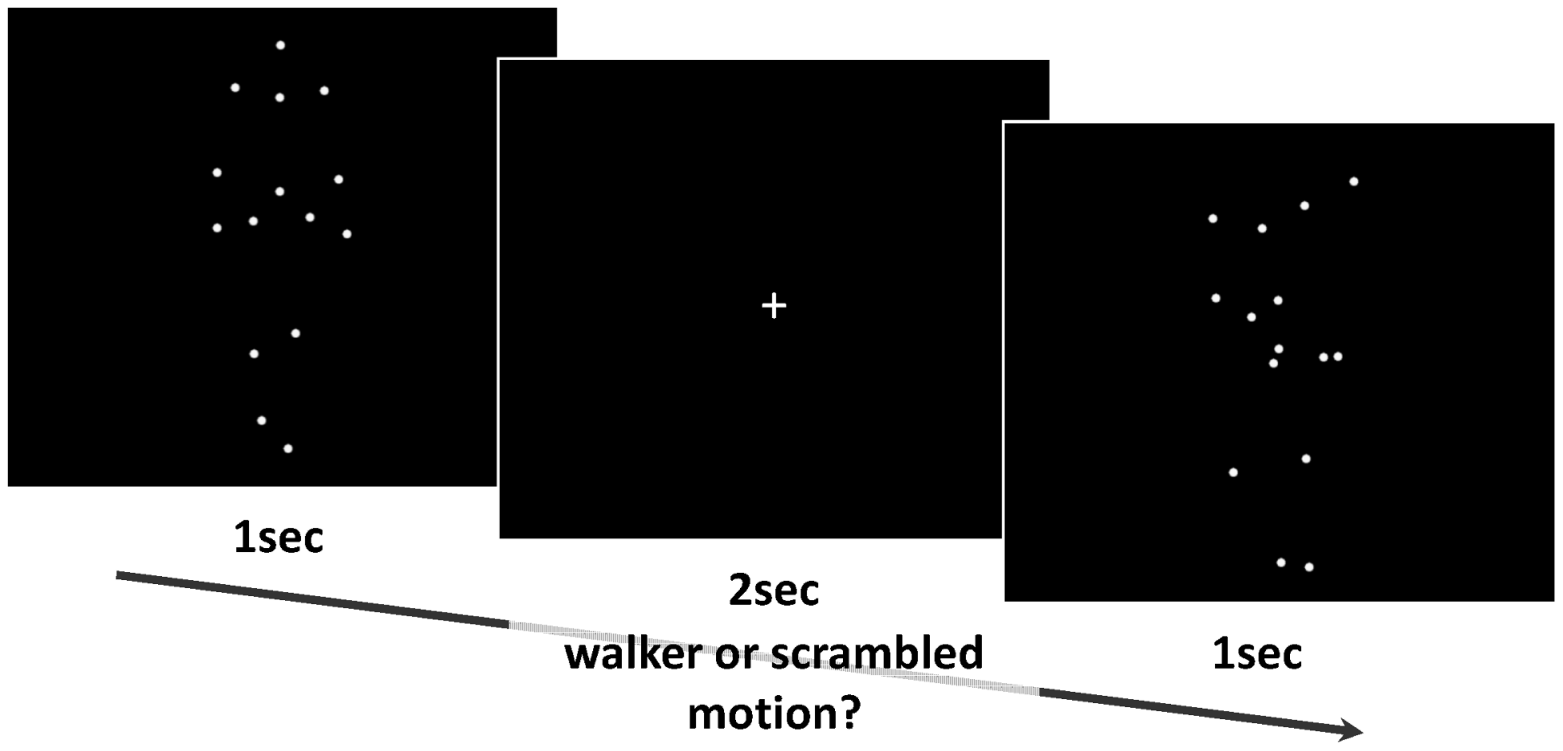
Example of experimental stimuli and stimuli presentation.

The biological motion stimuli (‘walker’ condition) consisted of moving point-light displays without contours. Fifteen female and 15 male walkers were used; each was marked by 15 white dots at the joints, tracking movements at the joints of the limps displayed against a black background. This was created using Labview version 6 (http//www.ni.com/labview). The walkers were shown in a frontal view walking with a speed of approximately two steps per second. Stimuli were based on motion capture data as previously described [48]. The ‘scrambled’ (i.e. control-) display was derived from these walkers by spatial scrambling. Thus the spatial position of the 15 dots was permutated while leaving the shape of each trajectory for each individual dot intact. This manipulation retains the individual frequency and acceleration profile of each dot, but masks the global acceleration profile indicative for biological motion. In sum, 30 different walkers and 30 scrambled motion stimuli were shown. Due to different starting points, dots were not strictly symmetrically organised. For the scrambled condition, dots were placed similar to the walker condition to cover the same visual field. Stimuli covered a visual field of about 12.5°×5.0° (height × width). Selected frames depicting both stimuli conditions are shown in Figure 1. Each individual stimulus was shown for 1 s, in a randomised order once per block. This block was repeated three times, thus each stimulus-class was presented 90 times. All stimuli were presented centrally on the screen. Between the stimuli, a white fixation-cross was presented for 2 s. In order to separate biological motion recognition and motor response, participants were instructed to react when the fixation cross (fixation period) appeared after the presentation of the stimulus. They were instructed to press the left mouse button if they had seen a walking person before and the right mouse button if they recognised a scrambled motion pattern (forced-choice). Correctness was emphasized over speed.

Children were allowed two breaks. The break duration was regulated by the participant. The experimenter continued the presentation after the participant's indication.

The stimulus sequence was controlled by PresentationTM software (http://www.neurobs.com/).

### Electrophysiological assessment

Continuous direct current EEG (DC-EEG) from 64 channels at a sampling rate of 500 Hz was recorded against a reference at Fpz using BrainVision MR-Plus amplifiers and Brain Vision Recorder software (Brain Products GmbH, Munich, Germany). An anti-aliasing low-pass filter with 250 Hz high cut-off was applied online. Sixty-four sintered Ag–AgCl electrodes (impedances<10 kΩ) were fixed by equidistant electrode caps (Easycap GmbH, Herrsching, Germany). Vertical and horizontal electro-oculograms (VEOG and HEOG) were recorded from electrodes placed 1 cm above and below the left eye and lateral to the outer canthi.

### Signal pre-processing

EEG-data was analysed using the Brain Vision-Analyzer 2 (Brain Products, Munich, Germany). Offline, raw EEG was high-pass filtered to 40 Hz (high cut-off) and recordings were transformed to an average reference. Continuous recordings were segmented into epochs of 3.5 s (starting 500 ms before stimulus onset until 3 s after onset). The first 500 ms of this epoch served as baseline. Only trials with correct responses were included in the analysis. The recordings were corrected automatically for eye movements and blinks by the algorithm of Gratton and Coles (Brain Vision Analyzer). Because no slow DC potentials were observable, no linear regressions to eliminate such trends were required. Artefacts were rejected automatically if the signal amplitude exceeded 150 µV (individual channel mode). This step was controlled by visual inspection, and remaining artefacts were removed by an experienced EEG technician who was blind to the study hypotheses. Electrodes with poor impedance or too many artefacts (in more than one third of all segments) were interpolated by nearest neighbours. The number of available artefact-free trials was compared between both groups. There were no differences between both typically developing controls (82.1±7.1) and ADHD (81.6±7.8) in the biological motion condition (t(40) = 0.23; p = 0.82), nor in the scrambled condition (typically developing controls: 80.9±9.7; ADHD: 78.8±10.7; t(40) = 0.68; p = 0.5).

### ERP analysis

**Figure 2:**
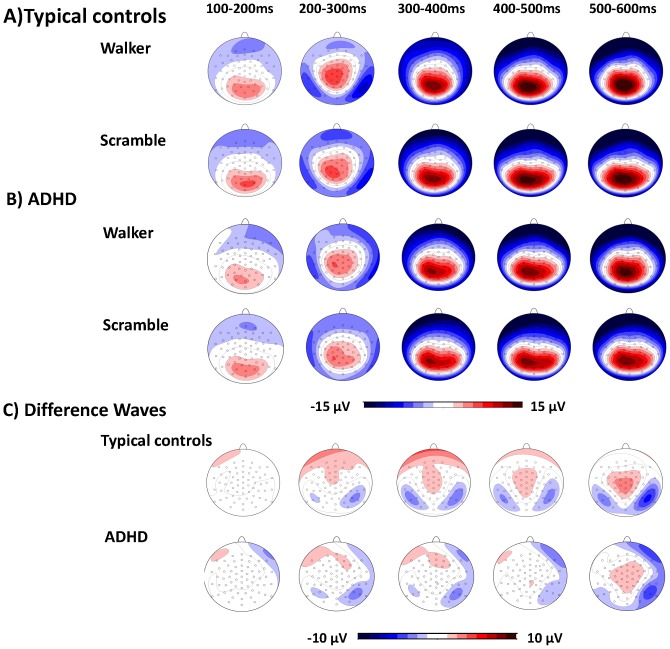
Topographic scalp maps. A) Maps for typically developing controls and B) for children and adolescents with ADHD in both experimental conditions and C) difference maps (walker – scramble) for both groups respectively.

Scalp regions and time windows of interest were defined according to previous studies and visual inspection of group grand averages of our data [23], [24], [31]: we determined the first positive peak (P100) between 100–200 ms at O1 (left hemisphere) and O2 (right hemisphere) and the first negative peak (N200) between 170–280 ms at P9 (left hemisphere) and P10 (right hemisphere) in each data set (see Figure 2 for topographical scalp maps). The latencies (ms) and amplitudes (µV) were exported for further analysis.

In some studies, a second negative component was observed [23], [31], [32], [49], but in our data, this second component was not observable on the scalp surface. For the slow positive deflection starting after 400 ms (P400+), mean activity at pooled centro-parietal leads (Pz, CPz, PO1, PO2, P1, P2) was assessed between 400–800 ms. Broader and later time windows for peak detection and mean activity calculations were chosen because the activity, especially in the late positive deflection, became prominent later in our data. This might be due to maturation differences in children and adolescents [50], [51].

### Analysis of dipole sources – biological motion specific activation

In order to further elucidate the cortical areas implicated in biological motion processing, we performed a spatio-temporal dipole source analysis using BESA research 5.1.8, Brain Electrical Source Analysis (BESA GmbH, Munich, Germany) according to Krakowski et al. (2011). First, we calculated the difference waveforms between the human motion and the scrambled stimuli in order to eliminate basic motion from biological motion processing.

In typically developing children, the difference waveforms indicated a stable topography with an occipito-temporal negativity and a central/centro-parietal positivity (see Figure 2) in good agreement with the topography described in adults for attended biological motion during the time-interval of 400–500 ms (Krakowski *et al.*, 2011). In contrast to Krakowski et al., the activation on our data peaked approximately 50–100 ms later. Therefore, we fitted two bilaterally symmetrical dipoles on the time interval 450–550 ms on the grand average of typically developing children, because of the better signal-to-noise ratio in grand averages compared to individual subject averages. Later time windows were not chosen, so as to minimize an overlap between the specific human motion activation and the P400+. Residual variance and dipole energy were minimized. Because the introduction of further dipoles did not further improve the model, the two-dipole solution was maintained.

Subjects with ADHD showed a more diffuse surface topography than typically developing subjects and lower amplitudes (see results section). Because of these low amplitudes, no reliable explanation of the surface topography was possible in the ADHD, as there were very high residual variances. Thus, no different source model was used for the ADHD group, but the source model fitted on typically developing children was applied to each individual subject. The fit procedure of the source model on the group grand average of typically developing children helped to minimize error variance that might distort dipole localization and/or orientation.

The dipole activations during the window of 450–550 ms after stimulus onset were exported for further statistical analysis in order to quantify the difference in the strength of the dipolar pattern reflected by the source model between groups.

In an additional, complementary approach to describe the more diffuse brain activity in ADHD, we also compared the amount of variance of the surface potential explained by the dipole model between groups. Accordingly, dipole orientations were refited for each individual subject's average and the explained variance (100%–residual variance) was exported for statistical analysis.

### Statistical data analyses

Statistical tests and correlation-coefficients were chosen according to the distribution of the dependent variables of interests. To test for group differences in percentiles of Raven matrices and handedness non-parametrical tests (Mann-Witney-U tests) were performed. Age, SCQ score and ADHD symptoms were compared between typically developing controls and ADHD by using a t-test. Group differences in number of correct responses and reaction times were tested with an ANCOVA with the within-factor MOTION TYPE (‘human-’/’scrambled motion’), between-factor GROUP, and age as covariate. Differences in ERP amplitudes and latencies were analysed by repeated-measures ANCOVAs with the within-factors MOTION TYPE and HEMISPHERE (right, left), the between-subjects factor GROUP, and AGE as a covariate. The P400+ was analysed by a repeated-measures ANCOVA with the within-factor MOTION TYPE, between-subjects factor GROUP, and AGE as covariate. Dipole moments in the right and left hemisphere obtained by source analysis were analysed by ANCOVAs with the factor GROUP, and AGE as covariate. The amount of variance of the surface potential explained by the source model was compared between groups by an ANCOVA with the between-subject factor GROUP, and AGE as a covariate.

Additionally we tested for differences between ADHD subjects with (N = 9) and without (N = 12) comorbid disruptive behaviour disorder in the ERP components or dipoles using t-tests. Furthermore, to exclude potential confounding effects of disruptive behaviour disorders, analysis between ADHD and typically developing controls were repeated but without the nine subjects with ODD or CD. In both approaches we focused at components, which differed between ADHD and typically developing controls in the ANCOVA models (N200 amplitude and dipole activation; see results section).

We further calculated exploratory Pearson correlations between SCQ score (for ADHD and typically developing controls respectively) and ADHD-symptoms (only ADHD subjects) with the N200 amplitude (averaged for both hemispheres and conditions) and the dipole activation (averaged for both hemispheres).

Significance levels were set to p = 0.05. The statistical analysis was performed using SPSS version 20.

## Results

### Sample

Descriptive data regarding age, handedness, IQ and SCQ score are summarized in Table 1. There were no significant differences regarding age (t(36.4)<1; p = 0.65), handedness (z(41)<1; p = 0.4) or Raven IQ percentile ranks (z<1; p = 0.5) between groups.

Similar to previous studies [4], [5], the ADHD group showed elevated SCQ scores (7.5±4.8) compared to typically developing control children (4.1±3.1; t(32.6) = −2.1; p = 0.01). ADHD children with ODD or CD (6.9±4.0) did not differ from the ADHD children without comorbid disruptive disorders (7.8±5.4) in regard to SCQ scores (t(18)<1; p = 0.67; see Table 1 for details). Also, both ADHD groups did not differ in ADHD attention deficits (ADHD with ODD/CD: 14.5±6.5; ADHD without ODD/CD: 16.3±5.1; t(18) = 0.7; p = 0.49) or hyperactive-impulsive symptoms (ADHD with ODD/CD: 19±11.5; ADHD without ODD/CD: 15.8±5.5; t(18) = −0.83; p = 0.42).

### Behavioural results

In the human motion condition, typically developing controls showed 94.2% correct responses and subjects with ADHD showed 94.8%. In the scrambled motion condition, typically developing controls showed 92.9% and ADHD 91.4% correct responses. There were no significant differences in the number of correct responses between the two groups (F(1,39)<1; p = 0.77) nor between both conditions (F(1,39)<1; p = 0.34; see Table 1 for details). Reaction times in both conditions (‘human motion’ and ‘scrambled motion’) also did not differ between the two groups (F(1,39)<1; p = 0.35) but all children reacted faster in the walker compared to the scrambled condition (F(1,39) = 4.8; p = 0.03).

### ERP analysis

**Figure 3:**
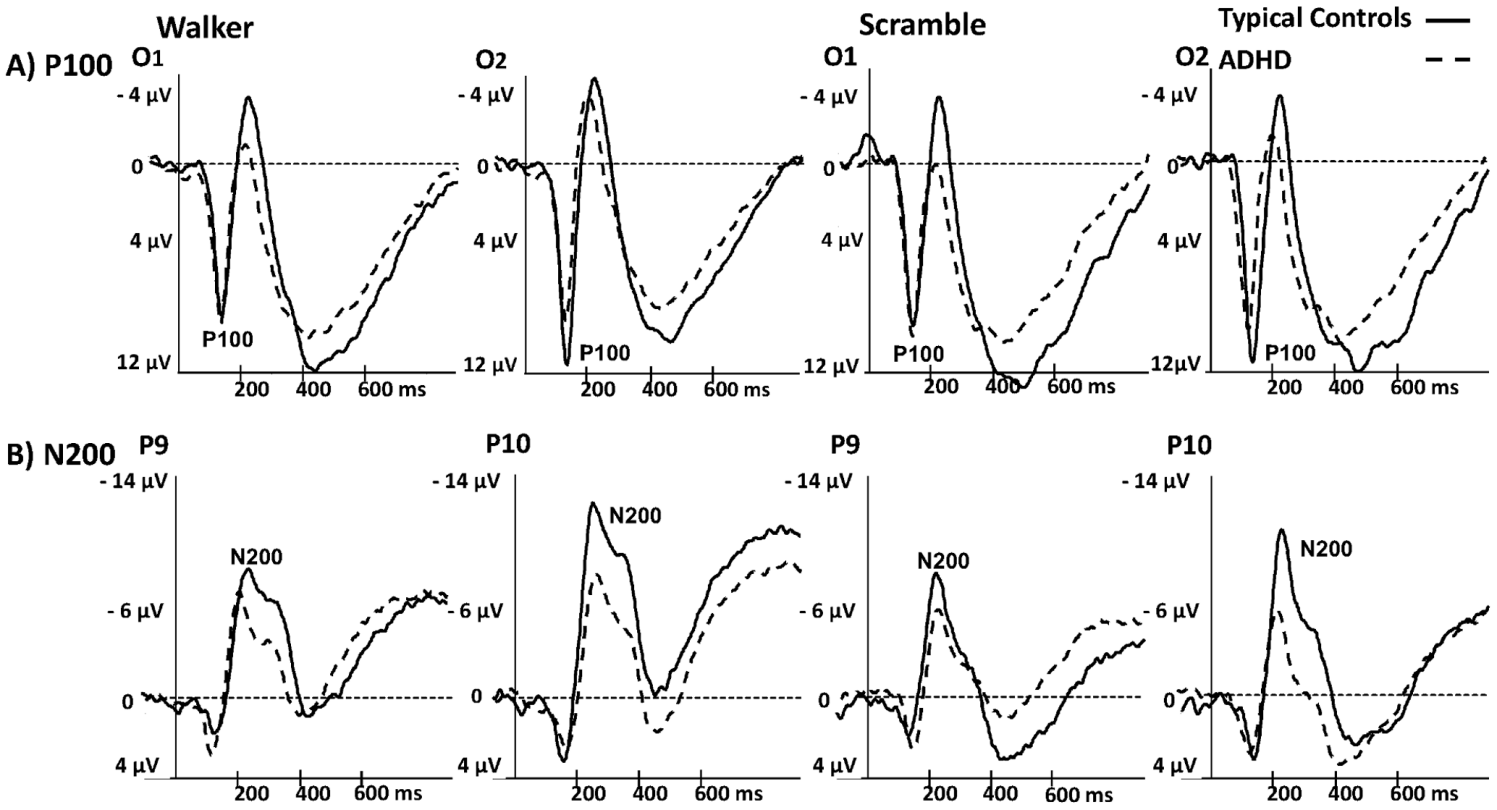
P100 and N200. A) P100 component at electrodes O1 and O2 and B) N200 at electrodes P9 and P10 for both conditions (walker, scramble) separately. Typically developing controls are indicated by solid lines and ADHD by dashed lines.

Regarding the P100 component, there was a negative association between age and P100 amplitude (F(1,39) = 10.2; p = 0.003; r = −0.46) as well as a trend for an interaction of HEMISPHERE*GROUP (F(1,39) = 3.3; p = 0.08). In typically developing controls, the amplitudes were slightly higher in the right hemisphere at O2, while in ADHD amplitudes were equally high at O1 and O2 (Figure 3). There were no significant effects regarding latency of the P100.

In respect to the N200 an effect of HEMISPHERE (F(1,39) = 6.0; p = 0.02) with higher amplitudes in the right hemisphere compared to the left, and an effect for GROUP (F(1,39) = 6.5; p = 0.01) with reduced amplitudes bilaterally in ADHD were observed. Furthermore, a trend for the interaction HEMISPHERE*AGE (F(1,39) = 4.0; p = 0.05) was also observable, however, neither the correlation between age and averaged activation in the right hemisphere (r = 0.17; p = 0.28) nor averaged activation in the left hemisphere (r = −0.17; p = 0.29) reached significance. There were also no significant effects regarding the latency of the N200 (Figure 3; all mean and standard deviations are presented in Table S1).

There were no significant differences in N200 amplitude (t(19) = 1.0; p = 0.34) between ADHD subjects with or without comorbid CD or ODD (see Table S2 for means and standard deviations). After excluding subjects with ODD or CD, N200 amplitude still differed by trend between ADHD (N = 12) and typically developing controls (F(1,30) = 3.2; p = 0.08).

**Figure 4:**
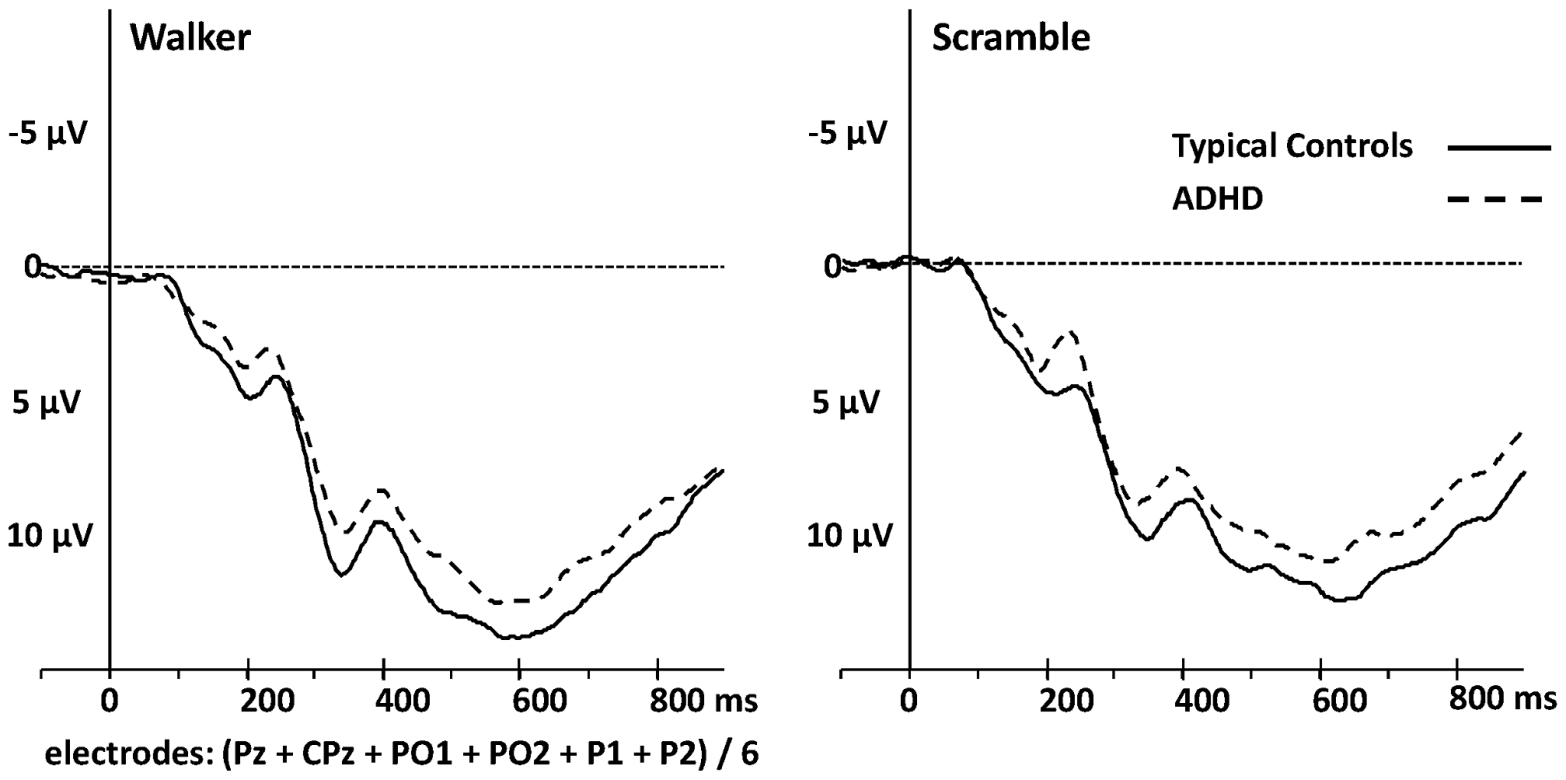
Late positive deflection. P400+ is shown at electrodes CPz, Pz, P1, P2, PO1 and PO2 (averaged) for the walker- and scrambled condition. Typically developing controls are indicated by solid lines and ADHD by dashed lines.

With regard to the P400+, no influence of MOTION TYPE, GROUP or AGE (including the interaction terms) was found (Figure 4; mean amplitudes and latencies are presented in Table S1).

### Analysis of human motion specific activation (human – scrambled motion)

**Figure 5:**
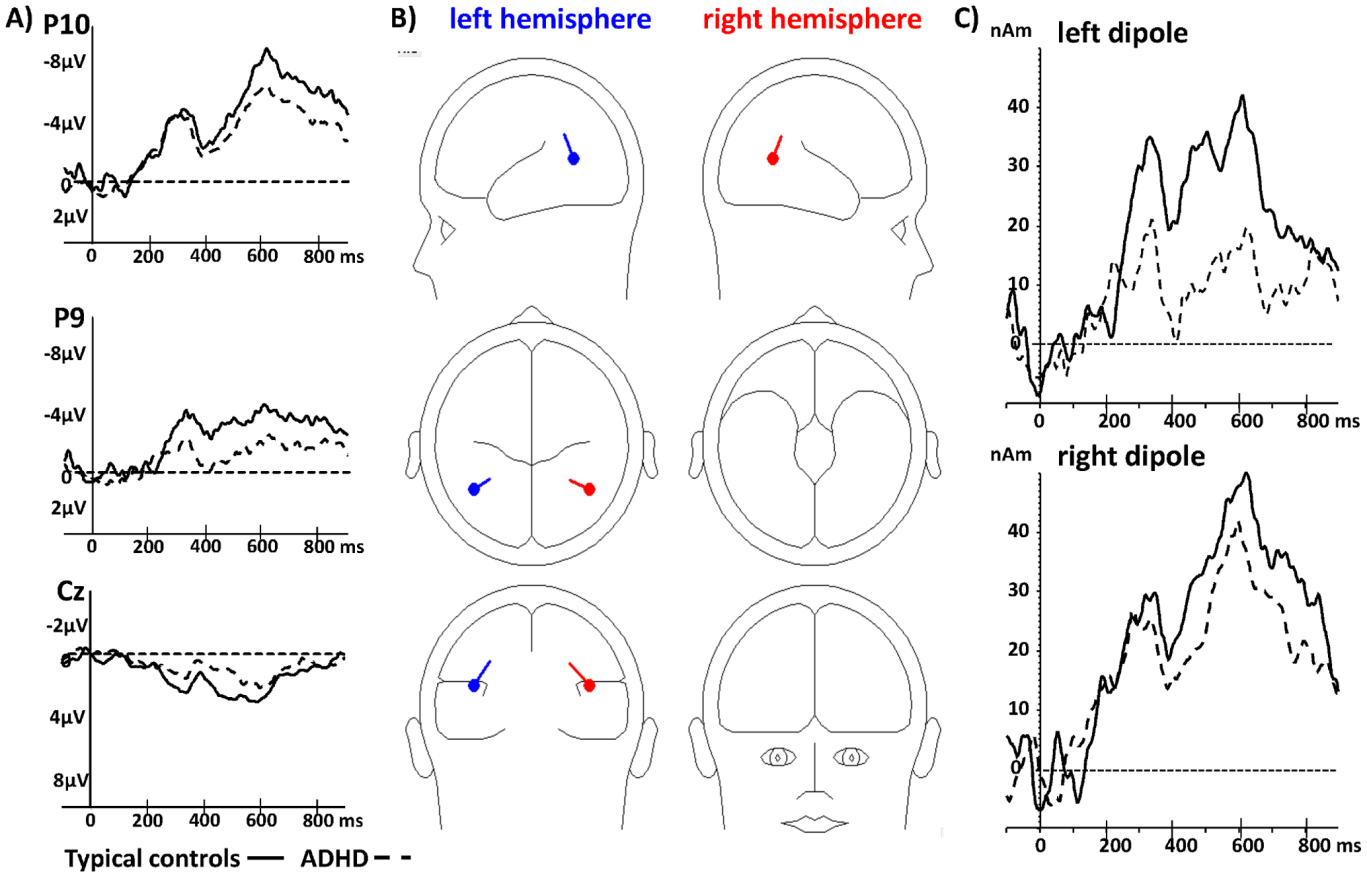
Dipole analysis. A) Difference waves at surface electrodes Cz, P10 (right hemisphere) and P9 (left hemisphere) in typically developing controls (solid line) and ADHD (dashed line); B) Dipole localisation in the left (blue) and right (red) hemisphere; C) Dipole waves, displaying activity in the left and right occipito-temporal junction in typically developing controls (solid line) and ADHD (dashed line).

The equivalent dipoles were located at the occipito-temporal junction, between the pSTS and MT+/V5. Both dipoles were located at a certain depth in the brain, indicating that they reflected the centre of gravity of larger active brain areas. Occipito-temporal negativity and mid-central/mid-centro-parietal positivity were explained by the same sources using volume conduction. The time-course of activity is shown in Figure 5.

There was a significant effect for GROUP (F(1,39) = 4.3; p = 0.04) and AGE (F(1,39) = 7.3; p = 0.01) with reduced activation in ADHD. The age effect mainly resulted from a negative correlation between age and the left dipole activation (F(1,39) = 7.2; p = 0.01; r = −0.40; see Table S1 for means and standard deviations).

The two-dipole model explained 88.5% of variance of the difference waves elicited by human vs. scrambled motion in typically developing controls, and 82.6% in ADHD. Also when dipole orientations were refit to single subject averages, the dipole model explained significantly less variance in the ADHD group (54.8±17) compared to typically developing controls (65.3±11.5; F(1,39) = 5.3; p = 0.03).

In addition, there were no differences between ADHD with or without comorbid CD or ODD in the left dipole activation (t(19) = 0.9; p = 0.38), nor in the explained variance of both dipoles (t(19) = 0.6; p = 0.53; see Table S2 for means and standard deviations). After excluding subjects with ODD or CD, dipole activation still differed between ADHD (N = 12) and typically developing controls (F(1,30) = 4.3; p = 0.05) and the group effect also remained significant (F(1,30) = 5.6; p = 0.03).

### Correlation trends for ADHD- and ASD symptoms and disturbed motion processing

We found no significant correlation between N200 amplitude and attention deficits (r = 0.22; p = 0.35) or hyperactive/impulsive symptoms (r = 0.18; p = 0.46). There were trends (without correcting for multiple comparisons) for the correlation between the dipole activation and attention deficits (r = −0.40; p = 0.08) and hyperactive/impulsive symptoms (r = −0.40; p = 0.08).

There were no correlations between the SCQ scores and neurophysiological parameters of human motion processing in typically developing controls (N200 amplitude: r = 0.18, p = 0.45; dipole activation: r = 0.12, p = 0.63) or in ADHD subjects (N200 amplitude: r = −0.15, p = 0.53; dipole activation: r = 0.20, p = 0.40).

## Discussion

The main aim of this study was to clarify if processing of biological motion is disturbed in children and adolescents with ADHD. We observed reduced activity in motion sensitive components (N200), to both biological and scrambeld motion, as well as a reduced activation in dipole sources which is specific for biological motion processing. Differences on the behavioural level were not found. In the following paragraphs, the results will be discussed in detail.

### ERPs

After about 100 ms a first positive deflection at occipital sites was observed. In typically developing controls, the amplitudes were slightly higher in the right hemisphere, while in ADHD amplitudes were equal in both hemispheres. Indeed, biological motion has been found to be processed predominantly in the right visual cortex [23], [24]. Thus a reduced lateralisation in ADHD could be an indicator of a reduced efficiency during visual processing of motion patterns. However, as this interaction only showed a trend, interpretations should be made cautiously. One previous study found clearly reduced occipital activation in ADHD during early perceptual analysis of facial expressions of anger and fear [12]. But in contrast to our stimuli, facial emotion expressions, especially of fear and anger, are also processed via the amygdala [52]. Thus, the reduced occipital activity in that study likely reflects a disruption of this early emotion-processing pathway between the amygdala and sensory areas and not deficits in visual processing in children with ADHD generally.

After 200 ms a negative deflection was observed at electrodes P10 and P9 – likely reflecting activity in MT+/V5 [27], with elevated amplitudes in the right hemisphere (P10). Our data clearly showed reduced activity of the temporal N200 in both hemispheres and in both conditions in ADHD. These reduced amplitudes might be due to attention related stimulus discrimination impairments, and deficits in stimulus evaluation which were described before in ADHD in an ERP study using an auditory oddball paradigm [53]. In contrast to the P100, during this second processing stage more attention might be needed to process all light-points and to detect motion coherence and motion direction. Furthermore, one study using magnetoencephalography showed that attention can affect these stages of biological motion processing in right temporal cortex [54]. Thus, children with ADHD might fail to allocate their attention focus properly at this stage of biological motion processing. On the other hand, we found no correlation between the N200 amplitude and attention deficits or hyperactive/impulsive symptoms, which might speak against such an association. But because we assessed those symptoms only via parent rating, it needs to be discussed whether a questionnaire is sufficient to assess such symptoms. Standardized behavioural tests might be better suited, to assess the real extent of attention deficits in a laboratory situation.

After 400 ms a slow and late positive deflection at parieto-central electrodes became evident. While earlier components reflect automatic processes, the P400+ is supposed to be a marker of a top-down, active cognitive process involved in decoding the stimulus content [24]. We did not find differences between ADHD and typically developing controls in this processing stage as both groups were equally capable in identifying the stimuli. This is likely due to a floor effect caused by the simple experimental task.

### Dipole sources – biological motion specific activation

We analyzed the difference waves between both conditions (‘human motion’–‘scrambled motion’) after 400 ms stimulus-onset by dipole source analysis. By comparing this difference, effects of non-biological motion processing are expected to be removed with the aim to investigate brain systems specifically involved in the detection of biological motion [31]. It is important to point out that those dipoles have to be interpreted as equivalent dipoles, i.e. not indicating an exact anatomical localization of brain activation, but a centre of gravity. Its strength is the temporal resolution, which allows differentiating unspecific early processing and later specific biological motion processing stages. Our source analysis approach (fitting a source model to the grand average of typically developing subjects and applying this model to all individual subjects) has the disadvantage that remaining error variance in the typically developing group grand average can increase dipole strength in this group. However, group grand averages have good signal to noise ratios and the method can be used to examine whether two groups show similar or different topographical activation patterns. Furthermore, when dipole orientations were refit to individual subject average data, the two-dipole-model explained significantly less variance in subjects with ADHD. Therefore the activation pattern was indeed less focused in subjects with ADHD, and the increased dipole strength in typically developing children and adolescents cannot only be explained by the fitting procedure.

Specific activations to biological motion were previously described in fMRI studies in the posterior temporal cortex, including regions also important for the processing and perception of social stimuli [13], [33]. Especially the right pSTS was found recently to be particularly sensitive for human motion compared to other biological motions, e.g. a walking animal [19]. Our dipole, approximately located in the occipital-temporal junction might therefore be centred in this region. The intraparietal sulcus which is located close to the dipole may also be indicated by the dipole, as it is involved in the detection of spatial relations [55], [56] which is also important for biological motion detection.

The ADHD group showed a significantly reduced activity after 450 ms post stimulus-onset in both dipoles. In addition, the variance of the cortex activation explained by both dipoles was lower in the ADHD group compared to typically developing controls. Interestingly, reduced or atypical activation in temporal regions in response to biological motion was observed in ASD before [57]–[59]. Thus it can be discussed if there might be similar processing abnormalities regarding social cognition difficulties in ADHD and ASD, which could be causal for ASD-like symptoms in ADHD [3]–[7]. Consequently, social interaction problems may need to be addressed adequately in ADHD therapy, at least in a subgroup of patients even without a formal categorical diagnosis of a comorbid conduct disorder or oppositional defiant disorder. However, social skills trainings like those recommended for high functioning ASD [60], [61] seem to have less effect on ADHD populations (see for a recent study [62] or review [63]). It also has to be mentioned at this point, that we found no correlation between ASD-like symptoms and the described processing deficits, although SCQ scores were increased in our ADHD sample, similar to previous studies [4], [5]. Therefore, mechanisms underlying this processing deficit might be independent from ASD-like symptoms in ADHD. Processing abnormalities did start in the MT+/V5 after 200 ms in ADHD. Deficits in the processing of motion direction and coherence may affect the subsequent processing of spatial relations and detection of biological motion indicated by the dipoles. During this late processing stage, stimulus information needs to be combined which could be difficult for persons with ADHD as earlier processing steps are already disturbed. Therefore, a reduced activation during the processing of biological motion might be caused by a deficient top-down attention allocation. However, future studies need to clarify if this processing deficit simply results from attention problems or indicates disruptions in higher-ordered processing networks.

### Behavioural data

Despite the observed differences on the neurological level we found no observable differences on the behavioural level – both groups were equally capable in identifying the stimuli. Such dissociations between neuronal activation and behavioural performance in biological motion perception were also found in ASD. For example in one fMRI study [64] subjects with ASD utilized different brain networks compared to typically developing controls but the behavioural performance was similar in both groups. Similarly, no differences in error rates were observed in another fMRI study [58] between ASD and typically developing controls despite differences on the level of neurological activation. Thus on the one hand it can be discussed that ADHD patients also use different networks to process these motion stimuli or that differences on the behavioural level can only be observed in more challenging tasks when the respective neuronal system breaks down. On the other hand ADHD children might simply need less “neuronal resources” to process biological motion because of excitability changes (see [65] for a discussion about the link between reduced brain activation and behavioural performance). Therefore it remains important to study the perception of biological motion in ADHD using different stimuli including distracters or noise to make these tasks more difficult or vary the level of intact and scrambled motion to identify different perception thresholds in children and adolescents with ADHD [64].

Another nice approach was adopted in one recent study [16]. Here, sensitivity in using global form or local motion cues for detecting facing or walking direction in point-light walkers was assessed. In this way it could be explored if subjects with ADHD may use different information sources to process biological motion. Interestingly only the ability to use form cues for biological motion processing was correlated with measures of social perception. Furthermore, we did not assess reaction times in our task in order to avoid artefacts in the EEG recordings. However, a longer reaction time could be an indicator of a higher cognitive effort to discriminate biological from scrambled motion [58] and should therefore be tested in further studies.

### Differences between ADHD with and without a comorbid disruptive behaviour disorder

We found no further differences between ADHD subjects with and without a comorbid disruptive behaviour disorder in the N200 amplitude and dipole activation. Furthermore, even after the exclusion of nine subjects with CD/ODD, the effects regarding the N200 amplitude and dipole activation remained robust. This indicates a stronger relation between ADHD and social processing deficits in this population than with comorbid disruptive behaviour disorders. Alternatively the negative results might be due to small sub-sample size. In our study, only nine ADHD subjects were diagnosed with a comorbid disruptive disorder. Larger sample sizes are needed to test for differences between these sub-populations and exclude confounding effects of comorbid disruptive behaviour disorders.

## Conclusion

In sum, for the first time we describe changes in biological motion processing in ADHD which starts 200 ms after stimuli onset in motion sensitive areas and continues at higher, more specialized processing stages. Thus our results suggest some alterations also in higher-ordered networks responsible for biological motion processing in ADHD. However, there are further open issues: first, we only included male participants, thus these results cannot be generalized to female populations. Secondly, the study sample size was still modest and the results need to be replicated in larger and also more heterogeneous samples (e.g. different comorbid disorders). Third, it needs to be addressed in future studies to which extend these disruptions are influenced by simple attention allocation deficits – and might therefore normalise in case of medication. Finally, it is important to point out, that as long as no deficits in the perception of biological motion stimuli are found it will remain unresolved whether the reduction of brain activity in ADHD really indicates a deficit in biological motion processing. Further studies need to address this question.

## Supporting Information

Table S1 **Mean amplitudes (µV) and latencies (ms) for all ERPs and mean dipole activation (nAm) separate for participants with ADHD and typically developing controls (TC).**
(DOC)

Table S2 **Differences between ADHD with CD/ODD (N = 9) and without (N = 12) regarding the N200 and dipole activation.**
(DOCX)
