# Supplementary material for: Visual Processing of Biological Motion in Children and Adolescents with Attention-Deficit/Hyperactivity Disorder: An Event Related Potential-Study
Source: PLoS One. 2014 Feb 10;9(2):e88585. doi: 10.1371/journal.pone.0088585 (PMC3919797; doi:10.1371/journal.pone.0088585)
Supplement: Table S1 — Mean amplitudes (µV) and latencies (ms) for all ERPs and mean dipole activation (nAm) separate for participants with ADHD and typically developing controls (TC). (DOC) [file pone.0088585.s001.doc]

**Table S1: Mean amplitudes (μV) and latencies (ms) for all ERPs and mean dipole activation (nAm) separate for participants with ADHD and typically developing controls (TC).**

|  | ADHD (N=21) | TC (N=21) | *statistics* |
| --- | --- | --- | --- |
| P100 Amplitude (μV) |  |  |  |
| *Walker* |  |  |  |
| O1 (±SD) | 9.67 (±4.13) | 10.32 (±5.47) |  |
| O2 (±SD) | 10.17 (±4.21) | 12.67 (±5.23) |  |
| *Scramble* |  |  |  |
| O1 (±SD) | 10.34 (±6.01) | 10.76 (±6.21) |  |
| O2 (±SD) | 10.47 (±5.82) | 13.13 (±5.76) | HEMISPHERE*GROUP F(1,39)=3.3; p=0.077 |
| P100 Latency (ms) |  |  |  |
| *Walker* |  |  |  |
| O1 (±SD) | 131.7 (±10.8) | 137.4 (±12.7) |  |
| O2 (±SD) | 128.9 (±7.8) | 135.5 (±15.6) |  |
| *Scramble* |  |  |  |
| O1 (±SD) | 132.2 (±9.1) | 133.7 (±12.9) |  |
| O2 (±SD) | 132.6 (±14.9) | 136.3 (±18.1) | no significant effects |
| N200 Amplitude (μV) |  |  |  |
| *Walker* |  |  |  |
| P9 (±SD) | -7.32 (±3.52) | -9.13 (±5.44) |  |
| P10 (±SD) | -8.97 (±4.76) | -13.05 (±6.96) |  |
| *Scramble* |  |  |  |
| P9 (±SD) | -6.47 (±3.30) | -8.50 (±4.39) |  |
| P10 (±SD) | -6.72 (±3.94) | -11.16 (±5.47) | HEMISPHERE F(1,39)=6.0; p=0.019  GROUP F(1,39)=6.5; p=0.014 |
| N200 Latency (ms) |  |  |  |
| *Walker* |  |  |  |
| P9 (±SD) | 220.4 (±15.1) | 225.3 (±20.7) |  |
| P10 (±SD) | 217.9 (±23.0) | 228.0 (±21.0) |  |
| *Scramble* |  |  |  |
| P9 (±SD) | 214.4 (±18.6) | 221.2 (±15.6) |  |
| P10 (±SD) | 219.1 (±18.6) | 225.9 (±14.6) | no significant effects |
| LPC Amplitude (μV) |  |  |  |
| *Walker* (±SD) | 10.95 (±4.34) | 12.33 (±6.05) |  |
| *Scramble* (±SD) | 9.81 (±4.48) | 11.14 (±5.45) | no significant effects |
| Dipole activation (nAm) |  |  |  |
| *left* (±SD) | 11.37 (±28.24) | 32.0 (±28.24) |  |
| *right* (±SD) | 25.26 (±26.25) | 33.89 (±25.41) | GROUP F(1,39)=4.3; p=0.044 |
