# Supplementary material for: Visual Processing of Biological Motion in Children and Adolescents with Attention-Deficit/Hyperactivity Disorder: An Event Related Potential-Study
Source: PLoS One. 2014 Feb 10;9(2):e88585. doi: 10.1371/journal.pone.0088585 (PMC3919797; doi:10.1371/journal.pone.0088585)
Supplement: Table S2 — Differences between ADHD with CD/ODD (N = 9) and without (N = 12) regarding the N200 and dipole activation. (DOCX) [file pone.0088585.s002.docx]

**Table S2: Differences between ADHD with CD/ODD (N=9) and without (N=12) regarding the N200 and dipole activation.**

|  | ADHD + CD/ODD | ADHD – CD/ODD |  |
| --- | --- | --- | --- |
| N200 Amplitude (μV) |  |  |  |
| *Walker* |  |  |  |
| P9 (±SD) | -5.62 (±2.35) | -8.59 (±3.79) |  |
| P10 (±SD) | -9.17 (±6.22) | -8.82 (±3.60) |  |
| *Scramble* |  |  |  |
| P9 (±SD) | -5.03 (±2.51) | -7.56 (±3.50) |  |
| P10 (±SD) | -7.20 (±5.35) | -6.37 (±2.67) | no significant effects |
| Dipole activation (nAm) |  |  |  |
| *left* (±SD) | 15.15 (±22.81) | 8.53 (±24.48) |  |
| *right* (±SD) | 31.79 (±17.82) | 20.37 (±31.0) | no significant effects |
